# Electromyographic activity of pelvic floor muscles in different positions during the use an innovative vaginal educator: Cross-sectional study

**DOI:** 10.1371/journal.pone.0291588

**Published:** 2024-03-27

**Authors:** Natália de Souza Duarte, Yury Souza De Azevedo, Emilly Cássia Soares Furtado, Lorena Jarid Freire De Araújo, Rayanne Mesquita Bendelack, Cibele Nazaré Câmara Rodrigues, Nazete dos Santos Araujo, Pablo Fabiano Moura das Neves, Ana Clara Nunes Soares, Rayana Carvalho Barros, Tainah Lacerda Santos, Erica Feio Carneiro Nunes, Elizabeth Alves Gonçalves Ferreira, Bianca Callegari, João Simão de Melo-Neto

**Affiliations:** 1 Institute of Health Sciences, Federal University of Pará (UFPA), Belém, PA, Brazil; 2 CAFISIO Mulher, Belém, PA, Brazil; 3 State University of Pará (UEPA), Belém, PA, Brazil; 4 University of São Paulo (USP), São Paulo, SP, Brazil; Public Library of Science, UNITED KINGDOM

## Abstract

The pelvic floor requires an integrated anatomical structure owing to its multiple functions. Therefore, it is necessary to study methods for improving muscle recruitment during training. This study aimed to analyze the effect of using an innovative vaginal trainer on the bioelectrical activity of the pelvic floor muscles. Pelvic positioning and interference factors, such as age, childbirth, sexual activity, urinary incontinence, and menopause, were also analyzed. A cross-sectional study assessed 30 women using an evaluation form, International Consultation on Incontinence Questionnaire-Short Form, and surface electromyography. The root mean square of a 5-second contraction period, peak root mean square values, area values, % maximal voluntary contraction (root mean square normalized by peak signal), and median frequency were collected. These findings with and without the use of a vaginal educator were compared in the anteversion, neutral, and retroversion pelvic positions. The use of a vaginal educator was found to increase the electromyographic activity of the pelvic floor muscles in the neutral position. In this position, older women showed an increased peak contraction when using the educator. Multiparas also benefited from increased bioelectric activity (root mean square and area). Sexually active women increased their bioelectric activity in a neutral position when using the trainer, exerting less effort in retroversion (%-maximal voluntary contraction). Incontinent and menopausal women exhibited slower body-building activation (decreased frequency) with the device, which requires further investigation. Our innovative biofeedback device induced greater recruitment of muscle fibers, is more effective in the neutral pelvic position, and may be effective in training the pelvic floor muscles, even in women with a greater tendency toward pelvic floor dysfunction.

## Introduction

The complex anatomical structure of the pelvic floor is composed of muscles, connective tissues, and nerves, allowing it to perform multiple functions, including urination, urinary continence, defecation, fecal continence, pleasure, and sexuality [1]. In addition, it supports the pelvic organs (vagina, uterus, anus, and rectum) and retains intra-abdominal contents [2]. Pelvic floor muscles (PFM) are composed of 70% slow-contracting fibers that provide an almost constant tone in this region [3].

Some authors believe that these muscles are less able to contract effectively to close the urethra, anus, and vagina and prevent leakage of urine and bowel contents when the pelvis is not in a neutral position [4]. However, other authors have found significantly higher tonic electromyographic activity of PFMs in the posterior pelvic tilt (retroversion) position [5]. Thus, the following question arises: "Are there differences in the electromyographic activity of PFMs as a function of the pelvic position?"

Certain factors predispose individuals to pelvic floor dysfunction (PFD), which can be divided into extrinsic factors, such as childbirth, and intrinsic factors, such as aging and climate [6]. Urinary incontinence (UI) is a common PFD that is classified according to the International Continence Society (ICS) as an involuntary loss of urine through the urethra and is considered a social and hygienic health problem [7]. This condition is considered to be one of the newest epidemics of this century, with aging being one of the main reasons for its occurrence and the female population being the most affected [8].

As women age, their sexual activity decreases. According to previous studies, women with a strong pelvic floor are more likely to report sexual activity and higher orgasm scores than those with a weak pelvic floor [9].

Even with the typical climacteric hormonal decline, postmenopausal women have lower PFM functionality. However, as a function of pelvic positioning, this group appeared to have greater bioelectric activity at rest and during exercise when in the posterior pelvic tilt position. Therefore, the hypotheses were as follows: "Can variables such as age, number of deliveries, UI, sexual activity, and menopause influence the bioelectric activity generated by PFM with and without the use of a vaginal educator?"

Pelvic floor muscle training (PFMT), electrical stimulation, and biofeedback are examples of therapies used to restore PFM functionality [10]. Because these therapies are effective and have few side effects, increasingly effective methods of administration have been investigated.

Teaching PFM contraction through verbal instructions is considered one of the most difficult tasks for physiotherapists [11]. However, previous studies have shown that biofeedback restores continence better than purely verbal instructions during PFMT [12].

Furthermore, the use of new technologies helps to advance the diagnosis and treatment of PFD and provides a better understanding of its pathophysiology [13]. Therefore, there is a constant need to develop innovative methods that encourage patients to receive complete therapy [14].

It should be noted that there is little innovation in the devices used to strengthen PFM through visual biofeedback. In addition, vaginal trainers currently available on the market are made of rigid materials, which can be uncomfortable for the patient and very expensive. Therefore, it is necessary to develop new devices with improved usefulness.

To date, no study has investigated the use of vaginal trainers. By knowing whether the use of a trainer can alter muscle recruitment, the optimal position to use it, and whether PFD influences this process, it can be inferred that its use is possibly relevant during PFMT by improving efficacy and providing faster PFD healing. Thus, this study aimed to analyze the effects of using an innovative vaginal trainer on the bioelectric muscle activity of the PFM in different pelvic positions and assess if variables such as age, number of deliveries, sexual activity, UI, and menopause influence the results. It was concluded that this innovative biofeedback device caused greater recruitment of muscle fibers, was more effective in the neutral pelvic position, and may be effective for PFMT.

## Materials and methods

### Ethical aspects

The study was conducted after approval by the Ethics and Research Committee of the Federal University of Pará (UFPA) (approval number 5.541.372) and signing the Informed Consent Form (ICF). The individuals were studied in accordance with the Declaration of Helsinki and the guidelines for research involving humans (Res. 466/12 and Res. 510/16 of the National Health Council guidelines).

### Study design

This was a cross-sectional observational study conducted in accordance with the Strengthening the Reporting of Observational Studies in Epidemiology (STROBE) Statement: Guidelines for Reporting Observational Studies (S1 Appendix).

### Setting and period of study

Data collection were conducted in a public maternal and child hospital in Belém, PA, USA. The study period was from August to September 2022.

#### Population

The participants were women aged ≥18 years. For the analysis, participants were divided into young (18–35 years), middle-aged (36–55 years), and elderly (≥56 years) groups [15].

### Sampling

Convenience sampling, or non-probability sampling, was the chosen sampling method.

### Sample size

For the sample size calculation, the interaction parameter related to the variable "intensity" was applied to the EMG amplitude, as described by Jeon *et al*. (2020) [16]. Partial eta-squared values (ηp2 = 0.640) and a minimal correlation between repeated measurements (r = 0.6) were used in this study [16]. Error probability values α of 0.05 and β of 0.2 were set for a number of 8 subgroups (with a non-sphericity correction of 1 for ANOVA) with repeated samples and with interaction between paired samples data. Thus, a minimum sample of 16 women was required; however, this study consisted of 30 participants.

### Eligibility criteria

**Fig 1:**
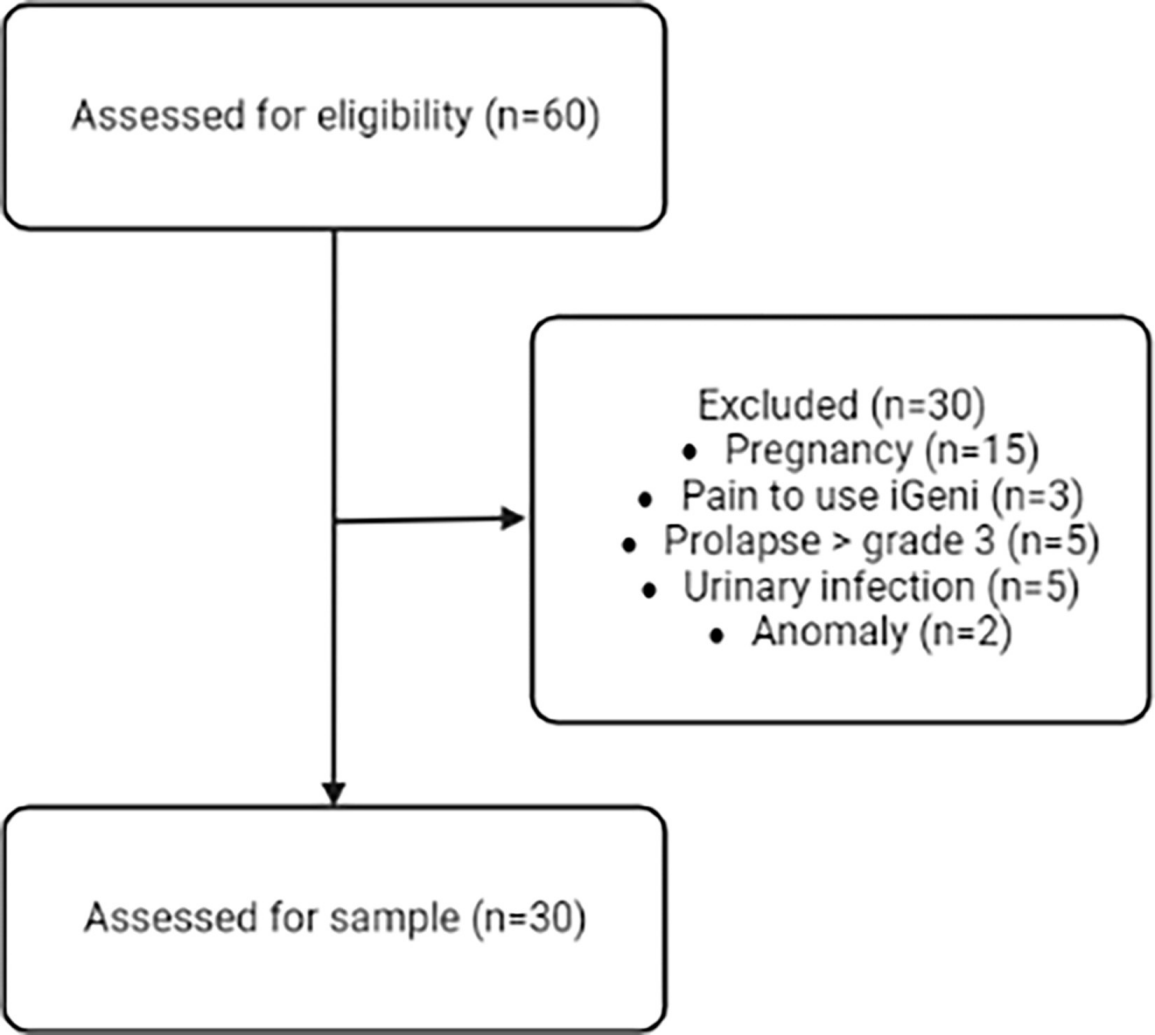
Flowchart of sample eligibility. Created by “BioRender.com” (2022).

Women aged over 18 years who agreed to participate in the study after signing the ICF were included. Women were excluded who were evaluated with muscle strength grade 0, vaginal prolapse grade ≥3 during vaginal inspection, pregnant women in the immediate postoperative period, diagnosed with neoplasia or undergoing oncological treatment, with neurological dysfunctions, urinary or vaginal infection, and pain during introduction of the vaginal educator, as well as those that presented incomplete data in the evaluation and abandonment form. A sample eligibility flowchart is described (Fig 1).

### Instruments and variables

#### Assessment form

Social, anthropometric, and clinical data were collected from all participants using an assessment form developed by the authors. The social variables collected were age (years), education (illiteracy, elementary school, high school, or higher), and family income (up to one minimum wage, between two and three minimum wages, between four and six minimum wages; and from 7 to 9 minimum wages). The Brazilian minimum wage was 1,212 reais.

Clinical variables collected were number of pregnancies, number of abortions, menopause (no menstruation for at least 1 year), active sex life, and presence or absence of urinary incontinence.

#### International Consultation On Incontinence Questionnaire—Short Form (ICIQ-SF)

The ICIQ-SF is a simple, brief, self-administered tool that can be used to quickly assess the impact of UI on quality of life and rank the patients’ urinary leakage [17]. In this study, a questionnaire was used to assess the presence or absence of UI. Women were classified as incontinent if they reported leaking urine at least once a week under the first question of the questionnaire.

#### Surface electromyography

Surface Electromyography (sEMG) is recommended for the real-time assessment of PFM contractions and functions by identifying the action potentials of motor muscle units [18]. The equipment used was the EMG System do Brasil®, which consists of a 4-channel analogue-to-digital converter with 16-bit resolution, an input range of -12 to +12 volts, a sampling rate of 2 KHz and a frequency range of 20 to 500 Hz. A computer was connected to the device, and the data were displayed at 2000 Hz. The electrodes (Medpex®) were self-adhesive and had a circular shape measuring 40 mm. They were composed of Ag/AgCl, and the solid hydrogel adhesive was composed of carboxymethylcellulose, glycol, and preservatives. Raw data provided by these devices were used for the preliminary analyses described in this study.

Although this study followed Surface ElectroMyoGraphy for the Non-Invasive Assessment of Muscles (SENIAM) guidelines, it should be noted there is no standardization or consensus regarding the pelvic floor area, which is the best electrode and acquisition site [19]. In this study, the electrodes were placed in the perineal area (lateral to the perineum), as shown in Fig 5; since the proposed device was inserted into the vaginal canal, it was not possible to use intracavitary electrodes [20]. Active electrodes were placed on the muscles at a distance of 20 mm, and the reference electrode was placed on the right malleolus peroneum.

The volunteers were instructed to perform a maximal voluntary contraction of the PFM for 5 seconds at each pelvic position (anteversion, retroversion, and neutral positions), first without and later with the vaginal educator. There was a pause of at least 30 s between each position, and the value was recorded with the device [21]. This method was chosen to reduce the risk of muscle fatigue since each patient was required to perform six contractions in different positions for a single measurement.

**Fig 2:**
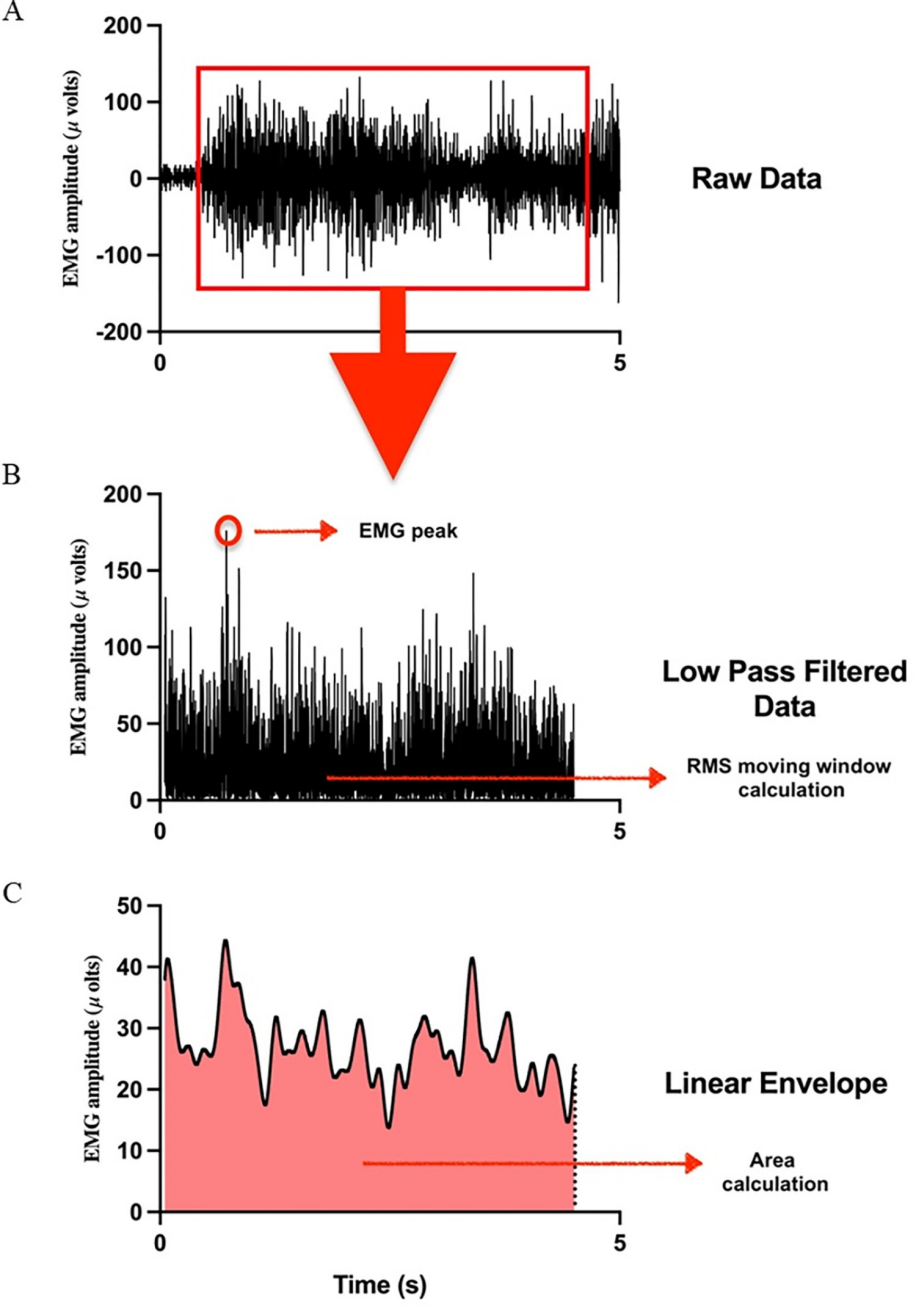
Graphic representation of the EMG signal treatment: raw (A), filtered (B) and enveloped (C).

The raw electromyographic signals were filtered between 20 and 400 Hz, full-wave rectified, and bidirectionally filtered using a 100-Hz low-pass Butterworth filter with zero delay. The root mean square (RMS) value was calculated using a 250-point window. After this step, a second-order low-pass Butterworth filter with a zero delay of 6 Hz was used for signal smoothing, and the EMG signal was integrated at the same contraction interval (0.5–4.5 seconds) (Fig 2).

The variables extracted from the signal were as follows: 1) RMS of the 5-second contraction expressed in microvolts (μV), 2) RMS peak values expressed in microvolts (μV), 3) area values (μV), 4) %-MVC (maximal voluntary contraction) (RMS normalized by the signal peak), and 5) median frequency of the signal after fast Fourier transform.

#### Vaginal educator

**Fig 3:**
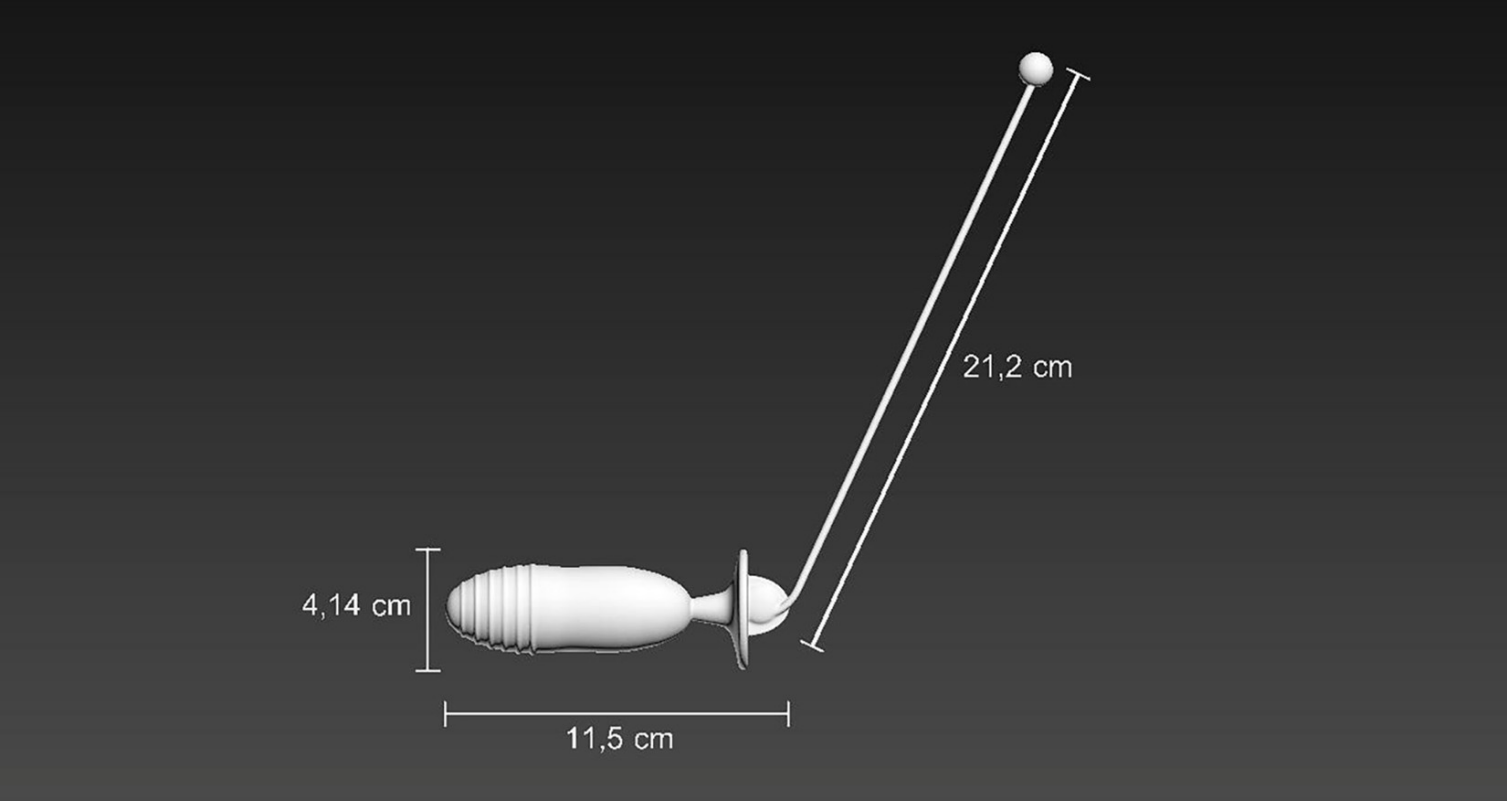
Schematic representation of the vaginal educator equipment.

The innovative vaginal educator weighed 500 g, had a diameter of 9 cm at the widest point, and a size of 7 cm for insertion into the vaginal canal with visual biofeedback using a 20 cm white antenna (Fig 3).

After insertion into the vaginal canal, the woman can see the antenna in front of her, which serves as visual biofeedback of her PFM contraction. The more the muscles are activated, the greater the backward tilt of the antenna. A vaginal educator is best inserted into the vagina using a unlubricated condom and water-based lubricant to improve comfort and avoid pathological conditions.

#### Data collection

Patients were recruited voluntarily through invitations on social networks and gynecology services at maternal and child hospitals. Those who met the eligibility criteria were invited to participate in the study and signed an ICF. After obtaining consent, the patients were instructed on the pelvic positions (anteversion, retroversion, and neutral position), how to perform maximal voluntary contraction of the PFMs, and the function of the vaginal educator device. After the volunteers were positioned in the lithotomy position, adhesive electrodes were placed on the perineal region at a distance of 2 cm, and the reference electrode was positioned on the external malleolus. Subsequently, the volunteers were instructed to perform a maximum voluntary contraction of the PFMs (with a verbal command for them to imagine holding urine and feces) for 5 s in each pelvic position (anteversion, retroversion and neutral), with at least a 30-second pause between each position, and the values were recorded by the devices [12]. After three rounds without the vaginal educator, the educator was inserted into the vaginal canal using an unlubricated condom and water-based gel, and the contractions were repeated in different pelvic positions. After data collection, patients received health education on PFM, were informed about their results, and were referred to a reference center for treatment if they had urogynecological complaints.

#### Bias

The study design made it susceptible to collection bias. To minimize this, data collection was repeated three times.

### Statistical analysis

These data are presented in S2 Appendix. Descriptive and inferential analyses were also conducted. Numerical variables were tested for normality using the Shapiro-Wilk test. For descriptive analysis, absolute and relative frequencies (%) were determined. The mean and 95% confidence interval (95% CI) were calculated. For the inferential analysis, a three-way repeated-measures ANOVA was performed to measure the effect size (F), p-value, and partial eta squared (ηp2). First, the isolated variables were compared in relation to the use or absence of the vaginal educator and among the three pelvic positions. They were then analyzed together and finally associated with other factors, such as age group, parity, sexual activity, UI, and menopause. The Bonferroni *post hoc* test was used for all variables.

### Outcomes

The primary outcome/dependent variable was the bioelectrical activity of the pelvic floor muscle in relation to the different factors analyzed.

## Results

The participant profiles are presented in Table 1. All data were self-reported. The volunteers had an average age of 45.16 ± 16.59 years, were predominantly housewives, had a high school degree, a family income of up to 1 minimum wage, were menopausal, active in sexual intercourse, and incontinent (Table 1).

**Table 1:** Sample characterization (n = 30).

Variables	Average	Standard deviation
**Age**	45,16	16,59
**Pregnancies**	2	1,92
**Abortions**	0,4	0,72
**Vaginal deliveries**	1,26	1,61
**Cesarean deliveries**	0,3	0,59
	N	%
**Occupation**		
Housewives	10	33,33
Retirement	6	20
Housekeeper	2	6,66
Other	12	40
**Education**		
Illiterate	1	3,33
Incomplete Elementary School	3	10
Completed Primary Education	8	26
Completed High School	11	36,66
Completed Higher Education	7	23,33
**Family Income**		
No Income	2	6,66
Up to 1 minimum wage	10	33,33
From 2 to 3 minimum wages	9	30
From 4 to 6 minimum wages	5	16,66
**Climacteric**	16	53,33
**Active sexual activity**	19	63,33
**Urinary Incontinence**	18	60

The comparison of the use of the vaginal trainer and the three pelvic positions analyzed separately did not show significant differences. However, when analyzing the interaction between the position and the use of the educator device, there were differences between the groups regarding the electromyographic parameters of RMS (p = 0.001) and area (p = 0.010) (Table 2). For both parameters, bioelectrical activity was significantly higher when the trainer was used in the neutral position (RMS [DM = 5.13; SE = 1.27]; Area [DM = 16.87; SE = 4.39]), confirming the hypothesis that the use of a vaginal educator alters the bioelectric activity of PFMs; however, this negates the hypothesis that the anteversion position would be the most effective (Table 3). Other interactions between the parameters of these two variables were insignificant.

**Table 2:** Comparison of bioelectrical activity in different pelvic positions and with and without the use of vaginal educator, with interference from the variables age group, number of deliveries, sexual activity and UI (n = 30).

Compare	RMS (μV)			Peak (μV)			Area (μV)			%CMV (μV)			Frequency (Hz)		
	F	ηp2	p-value	F	ηp2	p-value	F	ηp2	p-value	F	ηp2	p-value	F	ηp2	p-value
Pelvic positions	2,84	0,28	0,092^a^	2,59	0,46	0,105^a^	1,0	0,12	0,106^b^	2,82	0,17	0,078^a^	1,0	0,12	0,351^b^
Use of the vaginal educator	4,47	0,39	0,072^a^	2,55	0,26	0,154^a^	1,01	0,12	0,072^b^	3,44	0,20	0,086^a^	1,0	0,12	0,350^b^
Positions x Use of the vaginal educator	11,14	0,61	0,001^a^*	2,05	0,40	0,157^a^	1,01	0,12	0,010^b^*	1,01	0,12	0,144^a^	0,99	0,12	0,351^b^
Positions x Use of the vaginal educator x Age group	1,06	0,38	0,392^a^	0,84	0,45	0,267^a^	0,99	0,12	0,411^b^	0,99	0,12	0,424^a^	0,99	0,12	0,351^b^
Positions x Use of the vaginal educator x Number of deliveries	0,31	0,03	0,735^a^	0,03	0,00	0,969^a^	0,38	0,41	0,684^a^	0,34	0,03	0,710^a^	0,66	0,06	0,527^a^
Positions x Use of the vaginal educator x Sexual activity	2,01	0,16	0,167^a^	1,87	0,154	0,173^a^	1,82	0,15	0,182^a^	0,99	0,09	0,342^b^	0,03	0,004	0,963^a^
Positions x Use of the vaginal educator x UI	4,61	0,39	0,029^a^*	2,18	0,23	0,150^a^	3,86	0,35	0,046^a^*	0,79	0,10	0,473^a^	2,00	0,22	0,172^a^
Positions x Use of the vaginal educator x Menopause	2,36	0,15	0,114^a^	1,35	0,94	0,277^a^	2,31	0,15	0,118^a^	1,00	0,72	0,381^a^	4,22	0,24	0,026^a^*

Abbreviations: ηp2 (partial eta squared), RMS (Root Mean Square), %MVC (normalised maximal voluntary contraction).

a. Sphericity

b. Greenhouse-Geisser test.

**Table 3:** Comparison of bioelectrical activity with and without the use of a vaginal educator in different pelvic positions (n = 30).

		RMS (μV)				Peak (μV)				Area (μV)				%MVC (μV)				Frequency (Hz)			
Pelvic positions	Use of vaginal educator	Average	p	CI 95%		Average	p	CI 95%		Average	p	CI 95%		Average	p	CI 95%		Average	p	CI 95%	
				LL	UL			LL	UL			LL	UL			LL	UL			LL	UL
Anteversion	Without	19,13	0,461	14,21	24,04	93,53	0,741	78,73	108,33	60,91	0,460	45,02	76,80	0,20	0,187	0,18	0,23	139,63	0,904	124,79	154,46
	With	20,14		15,21	25,06	91,38		75,64	107,12	64,26		47,92	80,59	0,23		0,20	0,25	138,98		122,59	155,36
Neutral	Without	18,81	0,005*	13,95	23,67	94,88	0,068	76,60	113,15	59,72	0,006*	44,16	75,29	0,20	0,071	0,17	0,22	142,16	0,977	123,48	160,84
	With	23,94		18,35	29,53	101,77		79,63	123,92	76,59		57,52	95,67	0,22		0,20	0,24	142,40		125,48	159,41
Retroversion	Without	20,48	0,362	15,13	25,83	101,77	0,191	79,63	123,92	65,03	0,301	47,77	82,28	0,20	0,125	0,19	0,22	132,09	0,662	117,69	146,49
	With	21,75		16,45	27,05	119,56		83,51	155,61	69,74		52,26	87,23	0,19		0,17	0,22	134,32		118,65	149,98

Abbreviations: RMS (Root Mean Square), %MVC (normalised maximal voluntary contraction), CI (confidence interval), LL (lower limit), UL (upper limit).

*Bonferroni test.

Interactions with the third factor (age group, number of deliveries, sexual activity, urinary incontinence, and menopause) were analyzed.

The interaction between position × VE use × age group was not significant for all parameters (Table 2). However, in the *post hoc*-test analysis, young women showed higher PFM bioelectrical activity for the %-MVC parameter (DM = 0.03; SE = 0.01) in the anteversion position with an educator. In addition, older women had a higher peak (DM = 30.52, SE = 12.82) with the educator in a neutral position (neutral pelvic tilt) (Table 4).

**Table 4:** Comparison of vaginal educator use in each pelvic position by age group (n = 30).

			RMS (μV)		Peak (μV)		Area (μV)		%MVC (μV)		Frequency (Hz)	
Pelvic positions	Age group	Use of vaginal educator	Average (CI95%)	p	Average (CI95%)	p	Average (CI95%)	p	Average (CI95%)	p	Average (CI95%)	p
Anteversion	Young	Without	23,72 (12,39–35,06)	0,329	128,97 (81,66–176,28)	0,879	75,19 (38,55–111,82)	0,329	0,17 (0,13–0,22)	0,034*	134,25 (112,02–156,48)	0,063
		With	26,01 (15,10–36,93)		127,41 (83,73–171,08)		82,14 (46,61–117,67)		0,20 (0,16–0,24)		124,23 (100,82–147,63)	
	Middle aged	Without	15,70 (8,57–22,84)	0,891	66,33 (36,32–96,34)	0,636	50,37 (27,57–73,16)	0,891	0,24 (0,18–0,30)	0,387	155,07 (114,97–195,18)	0,286
		With	15,49 (8,42–22,55)		69,87 (35,09–104,64)		49,34 (26,78–71,91)		0,22 (0,18–0,27)		148,22 (116,81–179,64)	
	Older	Without	17,95 (12,16–23,75)	0,576	85,30 (51,12–119,48)	0,550	57,18 (38,71–75,65)	0,576	0,21 (0,18–0,25)	0,337	137,70 (113,30–162,10)	0,877
		With	18,91 (13,55–24,26)		76,87 (54,42–99,32)		61,28 (43,14–79,43)		0,24 (0,20–0,28)		136,33 (117,22–155,43)	
Neutral	Young	Without	27,86 (13,38–42,34)	0,321	142,82 (83,76–201,88)	0,987	87,54 (41,32–133,76)	0,321	0,18 (0,14–0,23)	0,167	122,50 (101,46–143,55)	0,471
		With	31,05 (20,16–41,94)		142,61 (87,35–197,88)		97,84 (62,10–133,58)		0,22 (0,18–0,27)		134,53 (93,95–175,11)	
	Middle aged	Without	14,89 (8,46–21,32)	0,057	74,29 (36,62–111,96)	0,293	47,71 (27,24–68,19)	0,057	0,21 (0,14–0,28)	0,946	147,80 (108,35–187,26)	0,274
		With	18,62 (11,72–25,51)		91,94 (50,49–133,39)		59,42 (37,39–81,44)		0,21 (0,16–0,26)		142,77 (104,99–180,56)	
	Older	Without	13,68 (9,33–18,02)	0,085	67,51 (43,70–91,32)	0,049*	43,92 (29,47–58,37)	0,085	0,20 (0,17–0,24)	0,422	156,56 (130,64–182,49)	0,077
		With	22,16 (11,81–32,52)		98,04 (55,95–140,12)		72,52 (36,78–108,27)		0,22 (0,17–0,27)		139,88 (115,57–164,18)	
Retroversion	Young	Without	28,35 (15,77–40,93)	0,649	152,81 (99,41–206,21)	0,364	89,23 (48,54–129,93)	0,649	0,18 (0,13–0,22)	0,483	120,42 (99,59–141,24)	0,930
		With	27,19 (14,49–39,89)		140,77 (91,46–190,07)		86,33 (45,35–127,30)		0,18 (0,14–0,22)		119,93 (97,59–142,26)	
	Middle aged	Without	15,33 (8,20–22,46)	0,693	66,63 (37,05–96,22)	0,249	48,92 (26,00–71,83)	0,693	0,22 (0,18–0,27)	0,381	148,28 (111,17–185,38)	0,802
		With	16,14 (9,46–22,82)		80,02 (47,93–112,11)		51,58 (30,27–72,89)		0,20 (0,14–0,26)		146,57 (111,09–182,06)	
	Older	Without	17,76 (15,23–20,28)	0,306	85,87 (49,08–122,66)	0,177	56,93 (49,04–64,83)	0,306	0,18 (0,14–0,22)	0,512	134,36 (114,74–153,99)	0,448
		With	21,92 (12,63–31,21)		137,89 (20,24–255,54)		71,32 (38,64–104,01)		0,21 (0,13–0,29)		129,08 (116,90–141,26	

Abbreviations: RMS (Root Mean Square), %MVC (normalized maximal voluntary contraction), CI (confidence interval).

*Bonferroni test.

The interaction between position and use of a vaginal educator × number of deliveries was not significant for all parameters (Table 2). However, in *post hoc* use, multiparous women in the neutral position showed greater bioelectrical activity with the educator for RMS (DM = 3.30; SE = 1.33) and area (DM = 10.50; SE = 4.29) and less for frequency (DM = -10.40; SE = 4.30) (Table 5).

**Table 5:** Comparison of vaginal educator use in each pelvic position by number of deliveries (n = 30).

			RMS (μV)		Peak (μV)		Area (μV)		%MVC (μV)		Frequency (Hz)	
Pelvic Position	Number of deliveries	Use of vaginal educatr	Average (IC95%)	p	Average (IC95%)	p	Average (IC95%)	p	Average (IC95%)	p	Average (IC95%)	p
Anteversion	Nulliparous	Without	23,70 (15,33–32,07)	0,535	123,40 (89,09–157,70)	0,445	75,50 (48,40–102,50)	0,551	0,18 (0,15–0,21)	0,084	136,70 (117,27–156,12)	0,675
		With	25,30 (15,69–34,91)		109,30 (70,52–148,07)		80,40 (49,79–111,00)		0,22 (0,19–0,26)		141,60 (108,76–174,43)	
	Multiparous	Without	15,60 (10,11–21,08)	0,915	104,50 (47,53–161,46)	0,442	49,20 (31,77–66,62)	0,987	0,18 (0,13–0,24)	0,429	134,50 (111,12–157,87)	0,661
		With	15,40 (9,92–20,87)		83,20 (41,68–124,71)		49,10 (32,26–65,93)		0,22 (0,16–0,28)		138,00 (114,84–161,51)	
Neutral	Nulliparous	Without	26,30 (14,22–38,37)	0,226	124,10 (79,09–169,10)	0,367	83,30 (45,27–121,33)	0,293	0,19 (0,16–0,22)	0,323	139,00 (110,83–167,16)	0,502
		With	29,60 (18,91–40,28)		137,40 (86,63–188,16)		92,90 (56,54–129,26)		0,21 (0,17–0,26)		147,90 (111,64–184,15)	
	Multiparous	Without	14,40 (9,83–18,96)	0,035*	83,60 (41,23–125,96)	0,351	45,40 (31,30–59,50)	0,037*	0,19 (0,15–0,23)	0,656	141,40 (113,97–168,82)	0,038*
		With	17,70 (11,72–23,67)		95,80 (53,99–137,61)		55,90 (37,24–74,55)		0,20 (0,16–0,24)		131,00 (110,58–151,41)	
Retroversion	Nulliparous	Without	27,10 (17,18–37,01)	0,960	146,40 (106,28–186,51)	0,584	86,20 (54,58–117,82)	0,951	0,17 (0,14–0,21)	0,502	127,50 (108,35–146,64)	0,765
		With	27,20 (17,09–37,31)		164,30 (76,14–252,45)		86,60 (54,06–119,14)		0,18 (0,14–0,22)		130,70 (105,09–156,30)	
	Multiparous	Without	16,10 (10,84–21,35)	0,678	83,90 (44,33–123,46)	0,511	50,70 (34,46–66,93)	0,626	0,21 (0,17–0,24)	0,052	129,50 (114,76–144,23)	0,725
		With	15,30 (10,21–20,38)		91,40 (58,76–124,03)		47,80 (31,79–63,80)		0,17 (0,13–0,22)		132,00 (117,48–146,52)	

Abbreviations: RMS (Root Mean Square), %MVC (normalized maximal voluntary contraction), CI (confidence interval).

*Bonferroni test.

The interaction between position × vaginal educator use × sexual activity was not significant for any parameter (Table 2). *Post-hoc* analysis (Table 4) showed that education led to increases in RMS (MD = 6.60; SE = 2.62) and area (MD = 21.49; SE = 8.49) for sexually active women in the neutral position. In addition, active women experienced a decrease in %-MVC scores (DM = -0.03; SE = 0.01) during retroversion with an educator. Finally, sexually inactive women had lower frequency scores for anteversion (MD = -9.02; SE = 2.85) and neutral positions (MD = -13.99; SE = 5.46) (Table 6).

**Table 6:** Comparison of vaginal educator use in each pelvic position per sexual activity (n = 30).

			RMS (μV)		Peak (μV)		Area (μV)		%MVC (μV)		Frequency (Hz)	
Pelvic position	Sexual activity	Use of vaginal educator	Average (CI95%)	p	Average (CI95%)	p	Average (CI95%)	p	Average (CI95%)	p	Average (CI95%)	p
Anteversion	Inactive	Without	16,23 (11,70–20,76)	0,337	74,09 (51,73–96,46)	0,499	51,72 (37,23–66,21)	0,335	0,22 (0,20–0,24)	0,528	150,32 (128,60–172,04)	0,010*
		With	18,20 (12,26–24,14)		81,18 (50,83–111,53)		58,23 (38,95–77,51)		0,22 (0,20–0,25)		141,27 (119,01–163,52)	
	Active	Without	16,50 (11,52–21,47)	0,872	91,62 (45,22–138,03)	0,513	52,68 (36,92–68,43)	0,896	0,21 (0,15–0,26)	0,666	140,41 (113,60–167,22)	0,656
		With	16,15 (11,42–20,89)		74,63 (48,85–100,40)		51,77 (36,89–66,65)		0,23 (0,17–0,28)		145,81 (119,65–171,97)	
Neutral	Inactive	Without	16,15 (10,51–21,78)	0,104	76,10 (52,30–99,89)	0,257	51,32 (33,41–69,23)	0,113	0,21 (0,17–0,24)	0,098	148,00 (124,23–171,78)	0,028*
		With	21,13 (12,48–29,79)		88,55 (53,94–123,15)		68,15 (39,21–97,08)		0,23 (0,20–0,25)		134,01 (117,85–150,18)	
	Active	Without	12,81 (8,79–16,88)	0,030*	65,25 (42,17–94,32)	0,246	40,80 (27,76–53,83)	0,030*	0,20 (0,15–0,24)	0,280	148,39 (118,30–178,48)	0,516
		With	19,42 (12,97–25,87)		87,08 (54,30–119,85)		62,29 (41,49–83,10)		0,22 (0,18–0,27)		160,74 (120,97–200,51)	
Retroversion	Inactive	Without	17,43 (13,56–21,30)	0,459	83,20 (58,01–108,38)	0,993	55,62 (43,38–67,87)	0,446	0,21 (0,18–0,24)	0,504	135,09 (120,78–149,41)	0,548
		With	19,58 (12,11–27,05)		83,11 (63,24–102,99)		63,38 (37,73–89,03)		0,22 (0,18–0,26)		133,00 (118,68–147,31)	
	Active	Without	16,51 (11,51–21,50)	0,976	70,73 (51,34–90,11)	0,141	52,39 (36,31–68,47)	0,983	0,22 (0,19–0,25)	0,022*	138,14 (111,70–164,58)	0,435
		With	16,45 (11,04–21,86)		86,75 (61,06–112,44)		52,26 (35,07–69,45)		0,18 (0,14–0,23)		147,59 (117,81–177,37)	

Abbreviations: RMS (Root Mean Square), %MVC (normalized maximal voluntary contraction), CI (confidence interval).

*Bonferroni test.

The interaction between position × vaginal educator use × UI was significant for the parameters RMS (without educator: 21.77 ± 6.50; with educator: 23 ± 7.05) and Area (without educator: 69.17 ± 20.53; with educator: 73.41 ± 22.37) (Table 2). However, the frequency scores in assessments with the educator were lower for women in the anteversion (urinary incontinence present: DM = -10.47; SE = 4.43) and neutral positions (urinary incontinence absent: MD = -13.24; SE = 5.19) (Table 7).

**Table 7:** Comparison of the use of vaginal educator in each pelvic position for IU (n = 30).

			RMS (μV)		Peak (μV)		Area (μV)		%MVC (μV)		Frequency (Hz)	
Pelvic position	IU	Use of vaginal educator	Average (CI95%)	p	Average (CI95%)	p	Average (CI95%)	p	Average (CI95%)	p	Average (CI95%)	p
Anteversion	No	Without	17,22 (9,18–25,26)	0,492	103,60 (33,92–173,29)	0,344	54,70 (29,262–80,14)	0,562	0,20 (0,14–0,27)	0,394	133,10 (100,99–165,21)	0,533
		With	16,05 (9,74–22,36)		70,67 (33,97–107,36)		51,52 (31,91–71,13)		0,25 (0,18–0,31)		138,98 (108,75–169,20)	
	Yes	Without	23,74 (11,92–35,56)	0,171	113,67 (63,93–163,41)	0,667	75,51 (37,45–113,57)	0,166	0,20 (0,17–0,23)	0,136	136,25 (113,95–158,55)	0,050*
		With	27,31 (15,98–28,63)		120,00 (74,71–165,30)		87,09 (50,88–123,29)		0,22 (0,20–0,24)		125,78 (102,01–149,55)	
Neutral	No	Without	13,55 (7,83–19,27)	0,182	70,97 (33,97–107,98)	0,761	43,12 (24,85–61,38)	0,187	0,20 (0,15–0,24)	0,088	150,38 (117,65–183,10)	0,038*
		With	17,89 (8,96–26,82)		73,66 (38,54–108,77)		57,35 (28,32–86)		0,23 (0,21–0,26)		137,13 (112,98–161,27)	
	Yes	Without	28,96 (14,02–43,89)	0,349	131,40 (72,27–190,52)	0,733	91,70 (44,34–139,07)	0,324	0,20 (0,17–0,24)	0,310	130,60 (99,28–161,92)	0,727
		With	31,74 (18,74–44,73)		135,97 (71,77–200,16)		101,10 (59,43–142,77)		0,24 (0,20–0,27)		137,05 (96,41–177,69)	
Retroversion	No	Without	18,07 (11,98–24,16)	0,111	77,41 (52,38–102,43)	0,465	57,62 (38,20–77,03)	0,101	0,22 (0,20–0,25)	0,096	126,20 (108,56–143,85)	0,342
		With	16,25 (10,02–22,49)		83,74 (48,15–119,34)		51,61 (32,00–71,22)		0,20 (0,17–0,23)		132,30 (114,74–149,86)	
	Yes	Without	29,10 (16,57–41,63)	0,871	143,71 (91,57–195,84)	0,394	92,40 (52,33–132,47)	0,925	0,19 (0,15–0,22)	0,431	117,69 (100,88–134,50)	0,916
		With	28,77 (16,09–41,45)		133,24 (82,68–183,79)		91,77 (51,16–132,38)		0,20 (0,17–0,23)		117,10 (97,26–136,95)	

Abbreviations: RMS (Root Mean Square), %MVC (normalized maximal voluntary contraction), CI (confidence interval).

*Bonferroni test.

The interaction between position, VE use, and menopause was significant for the frequency parameter (Table 8). Frequency scores (MD = -10.97; SE = 3.62) were lower for neutral-position educator use among menopausal women.

**Table 8:** Comparison of the use of vaginal educator in each pelvic position for menopause (n = 30).

			RMS (μV)		Peak (μV)		Area (μV)		%MVC (μV)		Frequency (Hz)	
Pelvic position	Menopause	Use of vaginal educator	Average (CI95%)	p	Average (CI95%)	p	Average (CI95%)	p	Average (CI95%)	p	Average (CI95%)	p
Anteversion	No	Without	16,54 (12,30–20,78)	0,457	76,90 (54,64–99,16)	0,961	52,91 (39,32–66,50)	0,426	0,22 (0,18–0,26)	0,419	147,70 (124,81–170,60)	0,904
		With	17,90 (13,54–22,23)		76,43 (55,62–97,24)		57,67 (43,39–71,95)		0,23 (0,21–0,26)		146,63 (123,68–169,59)	
	Yes	Without	21,00 (14,51–27,49)	0,615	121,38 (81,31–161,44)	0,425	66,67 (45,85–87,50)	0,619	0,18 (0,15–0,22)	0,314	131,55 (115,67–473,43	0,970
		With	21,81 (14,49–29,12)		104,78 (70,22–139,35)		69,21 (45,80–92,59)		0,22 (0,17–0,26)		131,32 (115,19–147,45)	
Neutral	No	Without	14,83 (11,00–18,65)	0,078	74,28 (51,35–97,21)	0,306	47,65 (35,34–59,96)	0,086	0,21 (0,17–0,25)	0,444	148,11 (107,94–174,06)	0,446
		With	19,36 (13,21–25,51)		87,16 (57,40–116,91)		62,79 (41,97–83,60)		0,22 (0,19–0,26)		159,57 (128,79–190,36)	
	Yes	Without	22,37 (13,48–31,26)	0,052	113,31 (74,84–151,79)	0,201	70,42 (42,14–98,71)	0,051	0,19 (0,16–0,21)	0,055	136,20 (115,77–156,64)	0,010*
		With	27,27 (19,21–35,32)		127,81 (89,92–165,71)		86,34 (60,33–112,35)		0,21 (0,18–0,24)		125,23 (109,22–141,25)	
Retroversion	No	Without	16,32 (12,57–20,07)	0,244	76,78 (52,23–101,33)	0,137	52,20 (40,09–64,31)	0,241	0,22 (0,19–0,25)	0,323	139,98 (118,27–161,70)	0,992
		With	19,28 (13,23–25,32)		110,22 (45,63–174,80)		62,47 (41,87–83,07)		0,21 (0,16–0,26)		140,07 (116,69–163,46)	
	Yes	Without	24,07 (16,68–31,45)	0,234	124,78 (88,89–160,67)	0,378	75,96 (52,28–99,64)	0,270	0,19 (0,16–0,21)	0,683	124,20 (111,42–136,97)	0,178
		With	22,57 (14,54–30,61)		117,40 (84,90–149,91)		71,61 (45,80–97,42)		0,18 (0,15–0,21)		128,56 (113,81–143,32)	

Abbreviations: RMS (Root Mean Square), %MVC (normalized maximal voluntary contraction), CI (confidence interval).

*Bonferroni test.

These data confirm the hypothesis that even in women who are more likely to develop PFD, such as older, multiparous, and incontinent women, the use of a vaginal educator can increase muscle recruitment in the neutral position.

**Fig 4:**
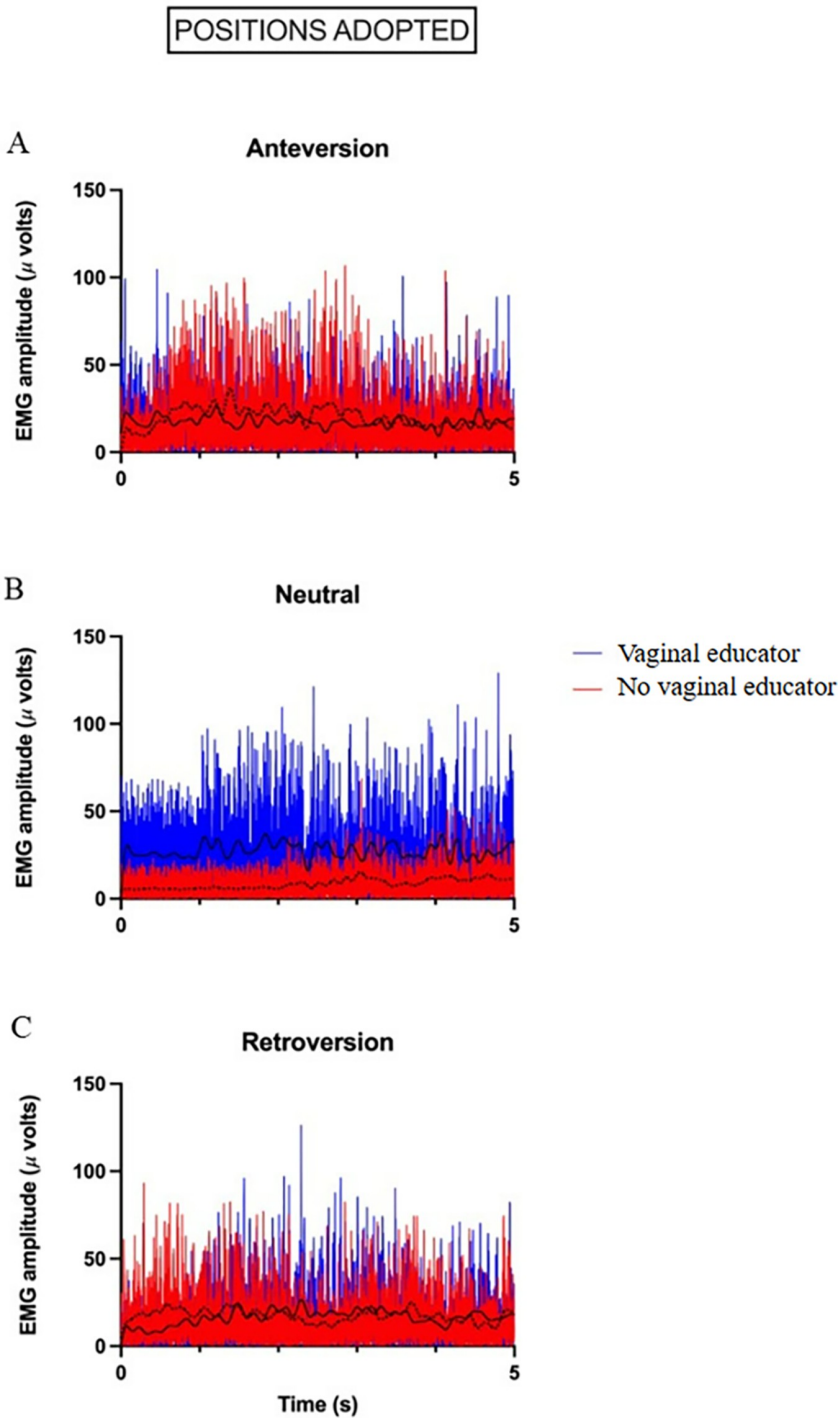
Filtered and rectified electromyographic signal from a representative participant in anteroversion (A), neutral (B), and retroversion (C) pelvic positions, with and without vaginal educator. The solid black line represents the linear envelope with vaginal educator and the dashed black line without vaginal educator.

**Fig 5:**
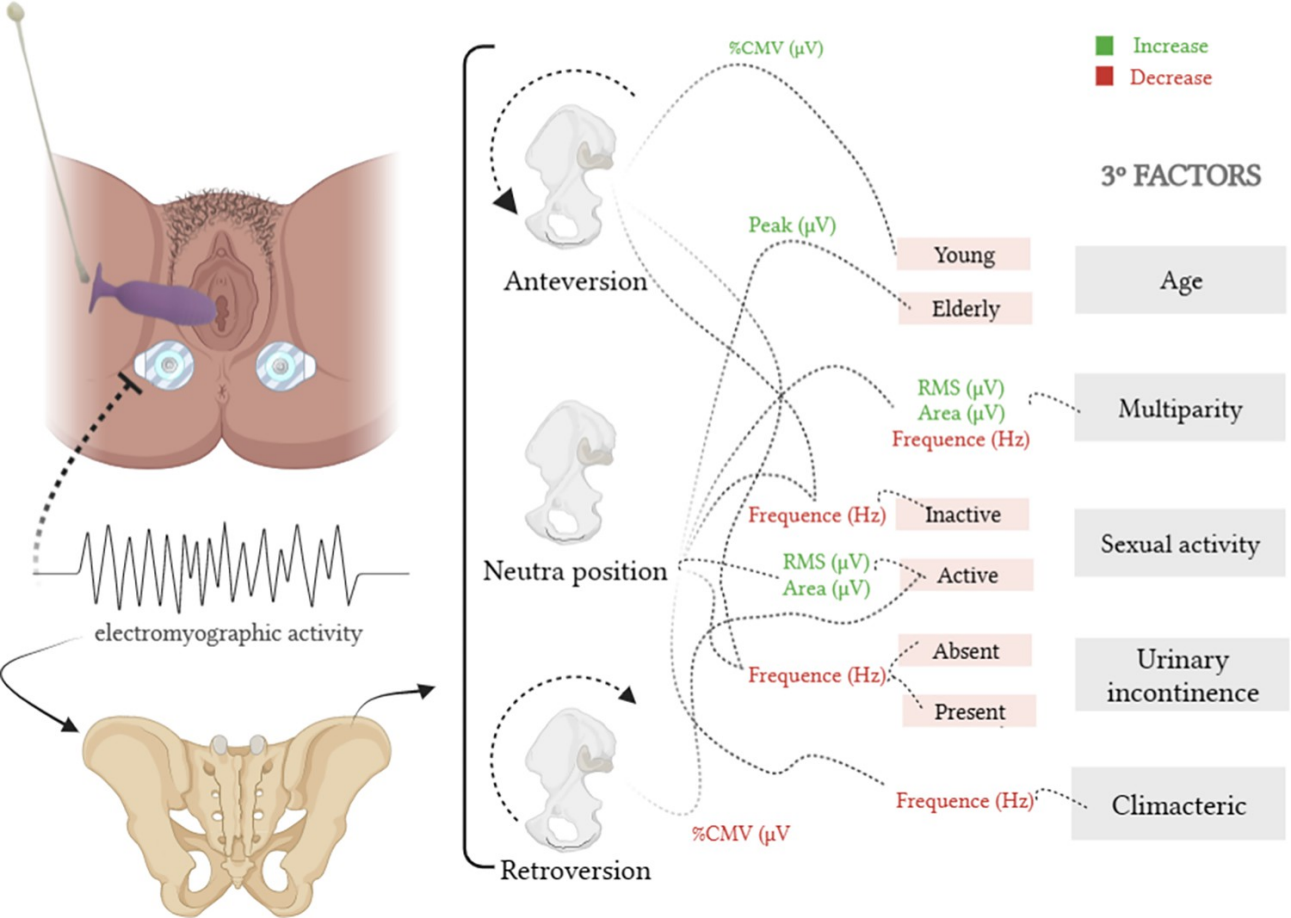
Mapping of the results found in the pelvic positions with the vaginal educator, in relation to the factors age, multiparity, urinary incontinence and climacteric, by “BioRender.com” (2022).

The electromyographic representation and mapping of the results are shown in Figs 4 and 5.

## Discussion

This study aimed to analyze the effect of using an innovative vaginal educator on PFM bioelectrical activity and asses whether differences between pelvic positions exist. In addition, the possibility of interference from variables such as age group, parturition, sexual activity, UI, and menopause was evaluated.

There is a correlation between muscle strength and the activation of motor units: the higher the electromyographic activity of a muscle, the better its function [22]. Therefore, electromyographic testing was considered the best option for achieving the proposed study objectives.

We investigated the acute effects of an innovative vaginal trainer for PFMT. Although no studies using vaginal educators were found, research using other forms of biofeedback associated with PFMT observed significantly greater increases in electromyography compared with PFMT alone, demonstrating the possible superiority of biofeedback integration [23].

The principle of biofeedback is based on reeducation through external feedback as a means of learning that allows patients to achieve increasing levels of contraction through visualization of a stimulus [24].

Our study showed in an acute manner that the use of a vaginal educator favors electromyographic activity in the proposed analyses. This finding indicates that women may benefit from visual biofeedback devices designed to improve PFM functionality.

When investigating the effect different pelvic positions had on the biomechanical benefit, participants showed greater RMS and area in the neutral position when using the vaginal educator.

According to Baracho (2018) [4], the neutral position favored PFM contraction, while other authors found significantly greater tonic electromyographic activity of the PFMs in the posterior pelvic tilt (retroversion) without the insertion of an instrument into the vaginal canal [5].

Retroversion favors the "closing" of the lower pelvis by bringing together muscle origins and insertions, reducing its length, and increasing its tension; thus, it is not even considered a good position in certain phases of labor [25]. In addition, this position favors the electromyographic activity of other muscles, such as the gluteus and multifidus, which may favor PFM activation [26].

Research has also found greater PFM activity at rest in the orthostatic position in a hypolordotic posture (which favors retroversion) than in normal and hyperlordotic postures [5].

Since the vaginal educator has some size and weight despite being anatomically designed for the vaginal canal, there may also be discomfort when contracting the PFMs in pelvic positions where there is greater pressure on the vaginal canal, thus favoring a neutral position during the use of the instrument.

In the present study, young women exhibited a higher %-MVC in the anteversion position with the assistance of an educator, suggesting a greater effort to achieve maximal contraction in this position.

Understanding the biomechanics of the hip/pelvis is important for evaluating PFM. In a study of elderly women (> 55 years) who performed only hip external rotation exercises without voluntary PFM contraction, a significantly greater increase in vaginal compression was observed, possibly because of the fascial attachment relationship between adjacent muscles [27].

It is well known that aging is a dynamic, progressive, and individual process that can be influenced by various factors. However, physical changes are expected in connection with this process, with emphasis on the decline of functional physical abilities such as flexibility and range of motion [28].

In addition, the increase in PFM stiffness that accompanies age negatively affects PFM function by decreasing muscle load, excursion, contractility, and regenerative capacity, which predisposes older women to pelvic floor dysfunction [29].

In the present study, older women in the neutral position with the vaginal educator had a higher contraction peak, which supports the use of the educator in this target group where a greater tendency toward PFM dysfunction is expected.

Along with age, parity heavily influences PFM activity. An animal study suggests that muscle damage occurs before and persists after birth [30]. As the number of pregnancies increases, the levator hiatus area increases, worsening the function of the PFMs [31].

In our study, multiparous women in the neutral position had higher electromyographic values for RMS and area parameters when using the device and lower values for frequency, suggesting that despite increasing muscle recruitment, it occurs slower. This may be the result of greater activation of type I muscle fibers.

One study found no differences in PFM electromyographic function and activity between nulliparous women of different age groups [32]. However, another study comparing PFM strength 45 days after vaginal delivery with that of nulliparous women reported a significant decrease [33].

Sexual activity also played a role in our analysis. The vaginal educator in sexually active women in the neutral position resulted in an increase in the RMS and Area parameters. In addition, there was a decrease in %-MVC values in retroversion with an educator, indicating that active women in this position required less contraction effort. Sexually inactive women had lower frequency values for the anteversion and neutral positions, indicating a lower rate of contraction.

Studies have shown that sexually active women and those with orgasms have better PFM resistance than nonsexually active women [34]. Furthermore, in pregnant women who are sexually active, there is a direct interaction between the frequency of sexual intercourse and the thickness of the levator ani muscle [35].

It is possible that this relationship is due to the fact that MAP is important to ensure adequate stimulation and arousal and to increase orgasmic potential [36]. This is because they exert involuntary activity in the form of rhythmic contractions during orgasm and increase vaginal sensation during penetration [37]. Therefore, better muscle function, which translates into better sexual function, may be a factor in the presence or absence of sexual activity. In addition to MAP, native vaginal tissue is essential for improving quality of life and sexual function [38].

Another important function of the pelvic floor is urinary continence, in which dysfunction can be observed even in adult female athletes [39]. Numerous resources have been studied, such as the use of novel digital therapeutic devices, extracorporeal magnetic innervation, and Kegel exercises, for the management of this condition, but a better understanding is still needed for the employability of tools for clinical implementation [40, 37D]; a principle which should also be applied to vaginal educators.

The frequency values when using the educator were lower in incontinent women in the anteversion position and in continent women in the neutral position. Women with incontinence have lower PFM activity, which worsens in the orthostatic position and is related to age and vaginal deliveries. The most common form of incontinence associated with muscle failure is stress urinary incontinence, which decreases electromyographic activity [41].

CH *et al*. (2005) [42] examined the effect of 12 weeks of biofeedback-assisted training on PFMs on women with stress and mixed urinary incontinence. They reported the muscle electrical potential increased from an average of 11.3 to 22 μV.

In addition, a study by Bertotto *et al*. (2017) [23] on postmenopausal women with stress urinary incontinence showed that eight sessions of PMT with biofeedback resulted in increased myoelectric activity. These findings suggest that using a vaginal educator in a longer treatment program may further improve our results.

Menopause was also analyzed in our study. In women, this biological event is primarily due to ovarian failure. It is detected 12 months after amenorrhea and generally occurs between the ages of 49 and 51 years. In this group, the frequency was lower when the vaginal educator was in the neutral position, demonstrating a slower rate of muscle contraction.

Due to changes in the female cycle, there is a loss of strength and function of the pelvic floor which is mainly observed in hormone deficiency and leads to PFD. These clinical and functional manifestations increase with age and after menopause.

The current study has some strengths, such as 1) the purpose of studying the vaginal educator, which is common in clinical practice but for which there is no scientific evidence; 2) adaptation of traditional educators to an educator with anatomical characteristics (size, format, and thickness); 3) verification of this type of device to activate the PFM; and 4) verification of the best pelvic positioning for better muscle activation. This study also had limitations, such as 1) this type of study does not allow for the verification of any chronic effects from using the device; 2) the sample of the study was from the Amazonian region, which may not be representative of other populations; and 3) voluntary participation may lead to self-selection bias in the results [43].

## Conclusion

The use of a vaginal educator has been shown to increase PFM electromyographic activity in the neutral position compared to verbal instruction alone and other pelvic positions. Volunteers likely to have lower PFM function, such as older, multiparous, and incontinent women, benefited more from the use of the vaginal educator in the neutral position than women likely to have better PFM function (young, nulliparous, and continent). Sexually active volunteers (likely to have better PFM quality) also show improved muscle recruitment when using a vaginal educator.

## Supporting information

S1 Appendix Strengthening the Reporting of Observational Studies in Epidemiology (STROBE) Statement: Guidelines for Reporting Observational Studies. (DOCX)

S2 Appendix Data of research. (XLSX)
