# Expression of Concern: Electromyographic activity of pelvic floor muscles in different positions during the use an innovative vaginal educator: Cross-sectional study

**DOI:** 10.1371/journal.pone.0360775

**Published:** 2026-10-19

**Authors:** The PLOS One Editors

The *PLOS One* Editors issue this Expression of Concern because this article [1] was identified as one of a series of submissions for which we have concerns about the integrity and/or reliability of the peer review process.

Readers are advised to interpret the article [1] with consideration of this issue. In following up on this matter, PLOS did not evaluate the article’s integrity or scientific validity.

PLOS has no evidence of any author involvement in the peer review concerns. The editor listed on the article is a PLOS Staff Editor who was not involved in the concerns, and who was unaware of the peer review issues before the article was published.
